# Post-Esophagectomy Hiatal Hernia: A Case Series

**DOI:** 10.7759/cureus.33214

**Published:** 2023-01-01

**Authors:** Vishu Jain, Subhash C Soni, Peeyush Varshney, Vaibhav K Varshney, B Selvakumar

**Affiliations:** 1 Surgical Gastroenterology, All India Institute of Medical Sciences, Jodhpur, IND

**Keywords:** paraesophageal hiatal hernia, surgical emergencies, post esophagectomy hernia, hiatal hernias, mie: minimally invasive esophagectomy

## Abstract

Post-esophagectomy hiatal hernia is a rare complication having varied presentation from asymptomatic cases detected incidentally on follow-up imaging to acute surgical emergency for strangulation or gangrene. Patients presenting as a surgical emergency have a prolonged post-operative course with significant morbidity.

We present three cases of post-esophagectomy hiatal hernia. Two of the three cases were operated for esophageal squamous cell carcinoma (SCC) and one patient was operated for esophageal leiomyomatosis. Two of the three cases (SCC and esophageal leiomyomatosis) underwent minimally invasive Mckeown’s esophagectomy and one case underwent robotic transthoracic Ivor-Lewis esophagectomy. All cases underwent contrast enhanced CT (CECT) and were biopsy proven prior to their index surgery. Both cases of SCC had prior neoadjuvant chemoradiation followed by surgery while esophageal leiomyomatosis underwent upfront surgery. All three cases have improved symptomatically and are doing well on follow up (case 1 - 12 months, cases 2 and 3 - 3 months).

All three of our cases have different clinical presentation in terms of symptoms, severity, and time duration from index surgery. Two of the three cases underwent emergency surgery and one case which was asymptomatic detected incidentally on surveillance imaging and was managed conservatively. Post-esophagectomy hiatal hernia is a rare entity with varying presentation. The management options in such cases vary depending on the severity of symptoms and time after index surgery. In cases presenting as surgical emergency, successful management depends on prompt detection, early surgery, proper post-operative care, and rehabilitation.

## Introduction

Hiatal hernia is an uncommon complication of esophagectomy. It can present incidentally, symptomatically, or as an emergency requiring urgent surgical intervention. The post-esophagogastric surgery hiatal hernia prevalence is 3.18%, and 2.01% of patients require surgical treatment [1]. Emergency repair is associated with higher morbidity (21% vs. 5.2%), higher mortality (5.5% vs. 0.65%), and longer hospital stay (8 days vs. 3 days) as compared to elective repair [2]. Symptomatic patients are managed with hiatal hernia repair with or without mesh placement. Asymptomatic cases are also prophylactically operated on; however, conservative management can be offered in some cases. We present three cases of post-esophagectomy hiatal hernia with different presentations and management approaches. 

## Case presentation

Case 1 

**Figure 1:**
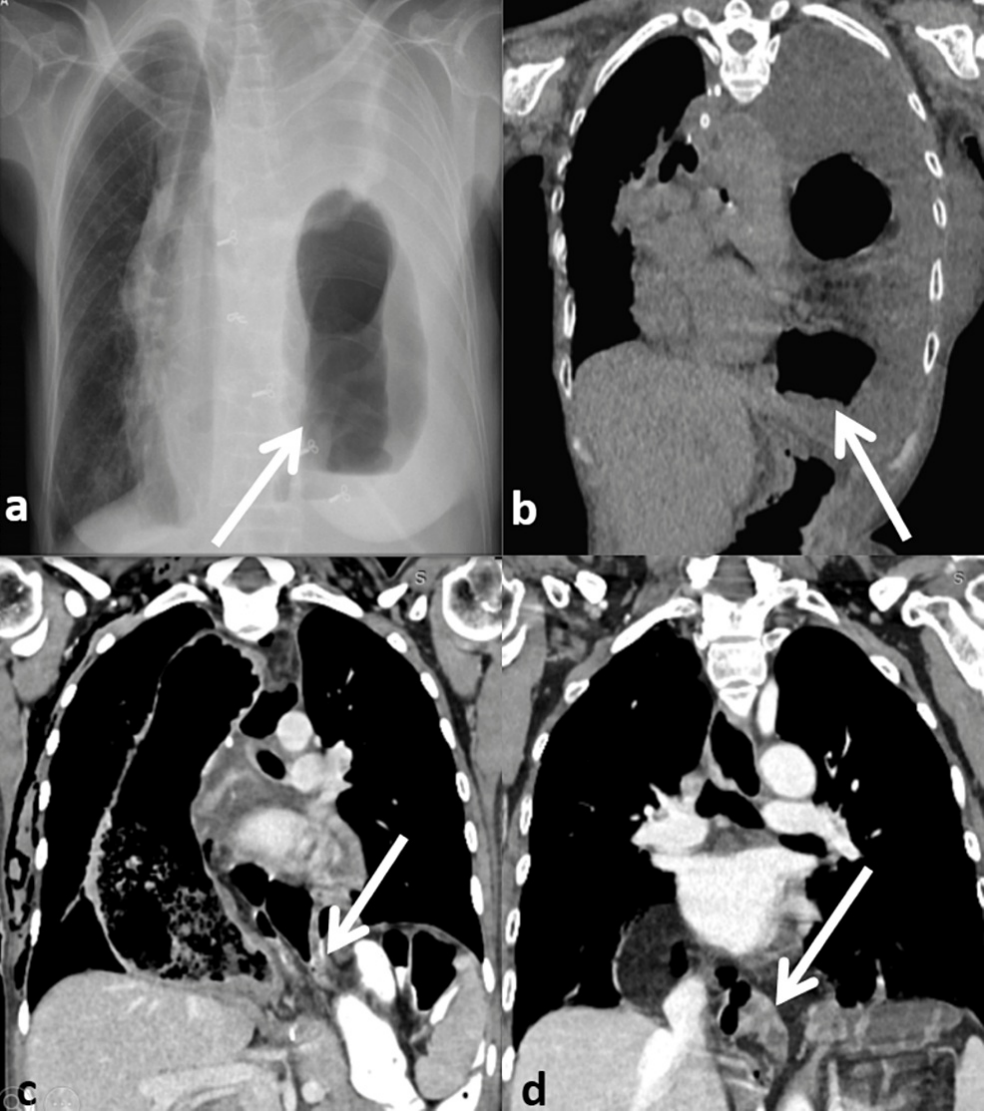
a and b - Chest X-ray and coronal view of HRCT chest of Case 1 respectively showing herniated dilated transverse colon loops with a compressed left lung and mediastinum shifted to the right. c and d - Coronal view of CECT of thorax and abdomen of Case 2 and Case 3 respectively showing herniated transverse colon loops. Solid white arrow shows herniated segment. HRCT, high resolution CT; CECT, contrast enhanced CT

A 62-year-old female with a body mass index (BMI) of 11.5 kg/m2 presented with complaints of severe abdominal pain, vomiting, decreased urine output, and difficulty with respiration for 3 days. She was a known case of squamous cell carcinoma (SCC) of the middle one-third of esophagus. She had received neoadjuvant chemo-radiotherapy (NACRT), following which she underwent a hybrid Mckeown’s esophagectomy with thoracoscopic mobilization of esophagus followed by laparotomy and gastric conduit formation and cervical esophago-gastric anastomosis 4 years prior. At the time of presentation, she had tachycardia, hypotension, and features of severe dehydration. There was tenderness but no abdominal signs suggestive of peritonitis or acute abdomen. On auscultation, air entry was absent on the left side. After receiving her in emergency, initially IV fluid was given and oxygen supplementation was started. Her routine blood investigations with arterial blood gas were done. and a chest X-ray was obtained. She was started on broad spectrum antibiotics. Her chest radiograph (Figure 1a) was suggestive of complete opacification of the left lung with right-sided tracheal deviation and mediastinal shifting with air-fluid levels on the left side of the chest suggestive of left diaphragmatic hernia with bowel herniation. A Ryle’s tube was inserted and urinary catherization was done and urine output was monitored. On evaluation, she had sepsis with pre-renal acute kidney injury which was managed expectantly with IV fluids and antibiotics. After resuscitation, a high resolution computed tomography (HRCT) of the chest and upper abdomen (Figure 1b) was done, which revealed a hiatus hernia with herniation of the transverse colon through it and twisting of the bowel loops at the neck of the hernia sac. The wall of the herniated transverse colon was papery thin with no wall enhancement suggestive of ischemia. Gross left-sided pleural effusion with collapse consolidation of the left lung was present. 

**Figure 2:**
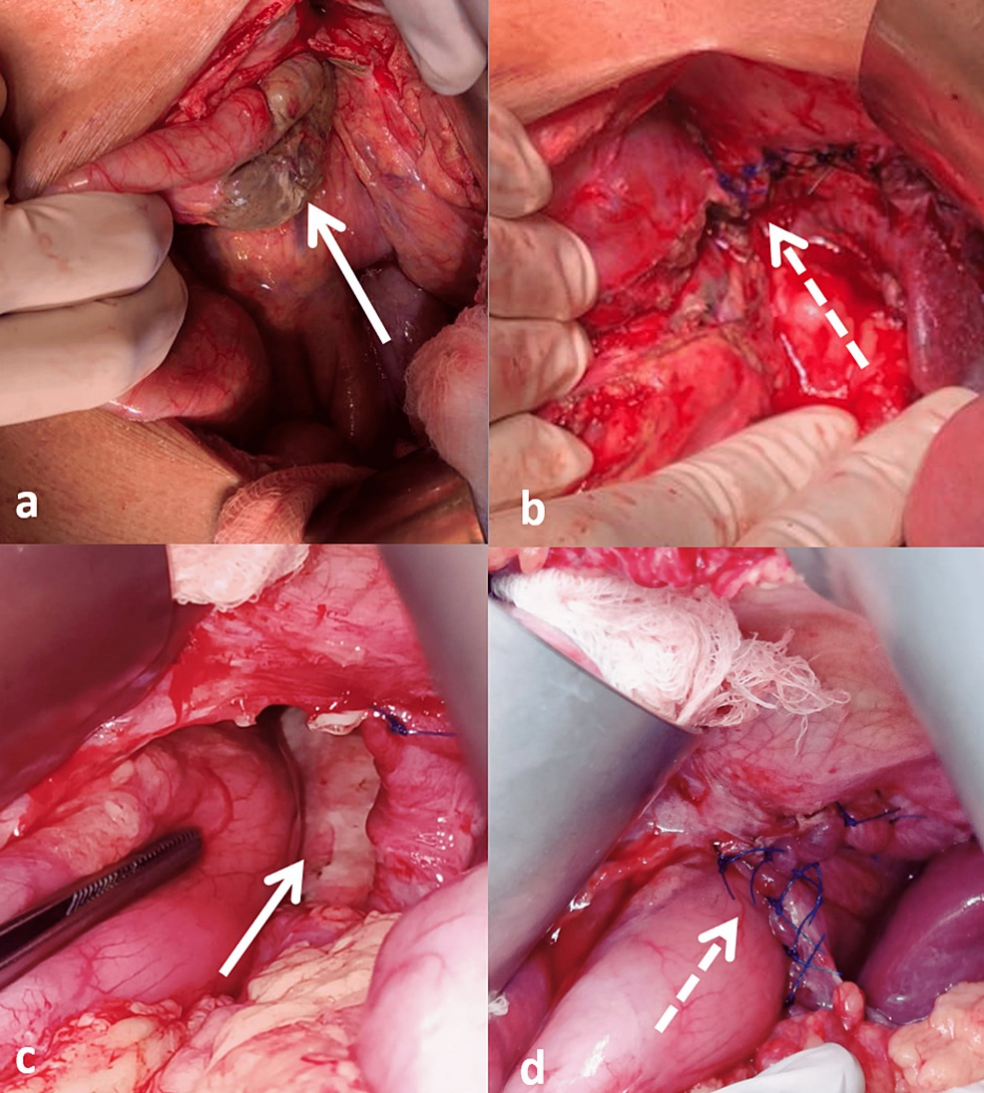
Intra-operative images: (a) the gangrenous transverse colon in case 1; (b) after resection of the gangrenous colon and repair of the diaphragm in case 1; (c) the hiatal hernial defect in case 2 (b) after suture repair of the defect in case 2. The solid white arrow shows the defect, the dotted arrow shows the suture line.

She then underwent exploratory laparotomy followed by reduction and resection of the herniated gangrenous transverse colon with primary repair of the hiatal defect and proximal ascending colostomy. Intraoperatively, there was a gross pleural effusion (2000 mL) present without any feculent peritoneal/pleural contamination. The esophageal hiatus was around 4 cm with a gangrenous transverse colon as the contents, present on the left side of the gastric conduit (Figure 2a). The distal limb of the transverse colon was rotated over the proximal limb, causing strangulation. However, the gastric conduit and rest of the abdominal organs were in the normal position. The hiatus opening was then widened and the gangrenous transverse colon reduced, resected, and a proximal ascending colon stoma with distal stump closure was performed. The widened hiatus was primarily repaired (Figure 2a,b).

Postoperatively, she had a prolonged ICU stay and underwent tracheostomy. She was then gradually weaned off the ventilator after establishing her enteral nutrition and shifted out of the ICU on post-operative day (POD) 39. She was then rehabilitated in the ward and was discharged on POD 46. Her tracheostomy was decannulated two months later. She underwent a restoration of bowel continuity three months later, which was uneventful.

Case 2

A 19-year-old female with a BMI of 15.2 kg/m^2 ^underwent minimally invasive esophagectomy for esophageal leiomyomatosis. A hybrid Mckeown’s esophagectomy with thoracoscopic mobilization of esophagus followed by laparotomy and gastric conduit creation with cervical esophagogastric anastomosis was done. A routine chest radiograph on POD 5 suggested herniation of the bowel loops in right pleural cavity with compression of the right lung and a mediastinal shift to the left. A contrast-enhanced computed tomography (CECT) of thorax and upper abdomen was done (Figure 1c), which was suggestive of a hiatal hernia with transverse colon as the contents, without strangulation or ischemia. She then underwent exploratory laparotomy and reduction of the herniated healthy transverse colon and omentum. The esophageal hiatus was 5 cm wide with a defect present by the left and anterior aspect of gastric conduit, which was primarily repaired (Figure 2c,d). She recovered well after the procedure and was discharged 5 days later. 

Case 3

A 60-year-old female with BMI of 22.9 kg/m^2 ^underwent total robotic transthoracic Ivor Lewis esophagectomy for SCC of the lower one third of esophagus after NACRT. Her post-operative period was uneventful. Her final biopsy showed complete pathological response. She was then on regular follow-up. A follow up CECT thorax and abdomen (Figure 1d) done at 6 months suggested herniation of the transverse colon and mesocolic fat into the posterior mediastinum adjacent to the gastric conduit. She was asymptomatic and is kept on expectant management.

## Discussion

The incidence of hiatal hernia post-esophagectomy is variable and ranges from 0.4% to 15% [1]. A meta-analysis by Bona et al. showed the post-esophagogastric surgery prevalence of hiatal hernia to be 3.18%, with 2.01% requiring surgery [1]. In our retrospective analysis of past 4 years, we have also found the hiatal hernia rates to be around 3%. The time to the development of hiatal hernia is also quite variable, ranging from 2 days to 12 years, although most cases present within 2 years of esophagectomy. In a meta-analysis of 15 studies by Hennesy et al., a total of 152 patients underwent surgical repair for post-esophagectomy hiatal hernia; 75 (49.3%) underwent elective repair (58 symptomatic and 17 asymptomatic) and 77 (50.6%) underwent emergency repair. This meta-analysis also included 99 patients with post-esophagectomy hiatal hernia who were asymptomatic, of whom 17 were electively repaired [3]. 

Some studies have reported a higher incidence of hiatal hernia in minimally invasive esophagectomy as compared to open surgery [1, 4]. Kent et al. in their study of 1075 esophagectomies had an incidence of 0.8 % in open cases vs. 2.8% in minimally invasive cases with an overall incidence of 1.86% [5]. Bona et al. in their study of 404 esophagectomies also showed a higher incidence of hiatal hernia in minimally invasive esophagectomy ranging upto 22.1% as compared to upto 12.3% after open procedures [1].

Risk factors associated with increased risk of hiatal hernia are female sex, minimally invasive esophagectomy, BMI less than 25 kg/m2, and extended hiatal opening. The rate of hiatal hernia also depends on the route of reconstruction used. The mediastinal route has a higher rate (0.4%-4% vs. 1%) as compared to the retrosternal route because the hiatal widening is done using the mediastinal route to prevent obstruction [6]. A few authors have also recommended fixation of the gastric conduit to the hiatus to prevent hiatal hernia. However, this creates an additional risk factor for damaging the vasculature of the gastric conduit [7]. All three of our patients had all these risk factors, including minimally invasive surgery, female sex, and a low BMI. The reason for low BMI in all of our patients could be due to serving population which belongs to the poorer economic strata of the society who already have a low baseline nutrition. Additionally, case 1 was an elderly lady with a small and slim body habitus.

In a study by Narayanan et al., the use of neoadjuvant therapy was also found to be associated with an increased incidence of diaphragmatic hernia, which may relate to more extensive perihiatal dissection required in patients with more advanced diseases who receive chemo-radiation [8]. It has also been observed that left-sided hiatal hernia is more common than on the right side, with 87%-100% of cases occurring on the left side [1, 5, 9]. One possibility is the staple line along the lesser curvature of the stomach and smooth serosal surface over the greater curvature of the stomach causing more adhesion formation over the right crus. The lateral edge of the left lobe of the liver also acts as a mechanical barrier in the right crural region, possibly preventing a right-sided hernia [9].

Management usually depends on the type of presentation and severity of symptoms. Most of the time, emergency surgery is required for acute presentation, but a hiatal hernia may be picked up incidentally during routine or follow-up imaging for malignancy. Bona et al. reported 57.2% of cases requiring emergency repair, among whom 6.9% required bowel resection as well [1]. The rest were elective repairs done by minimally invasive, hybrid, or open techniques. Conservative management has also been attempted successfully by Willer et al. and Brenkman et al. in 2 and 19 cases, respectively [10-11].

Clinical presentation varies from asymptomatic in incidentally detected patients to severe respiratory distress. Patients may also have abdominal complaints like dyspepsia, dysphagia, constipation, vomiting, and regurgitation. On chest auscultation, decreased air entry on the side of the hernia along with a fall in oxygen saturation aids in clinching the diagnosis, especially in emergency situations. On abdominal examination, there might be signs of peritonitis in cases of perforated viscera. Diagnosis of diaphragmatic hernia post-esophagectomy is clinically difficult and requires a high level of suspicion. 

A chest radiograph is the first investigation followed by CECT of the thorax and upper abdomen; this has good sensitivity and gives excellent anatomical details on the contents of the hernia and the viability of the contents, such as the bowel. Early CT should be done to avoid delay in diagnosis as there might be strangulation and perforation of bowel which are associated high risk of mortality, if not treated promptly. 

As per the Society of American Gastrointestinal and Endoscopic Surgeons, asymptomatic hiatal hernias can be conservatively managed depending on the patient’s age and comorbidities. However, all symptomatic patients should be offered surgical management [12]. The presence of severe hiatal hernia-related complications, such as incarceration, ischemic bowel complications, strangulation, or bowel perforation, are indications for emergency surgery. Complicated hiatal hernias are associated with high rates of mortality. There has been only one reported case of the successful management of prolapsed transverse colon rupture after esophagectomy, by Konno-Kumagai et al. [13]. 

Surgical options include reduction of the contents and primary repair of the defect. Bowel resection may also be required in around 7% of such cases, and a primary anastomosis or stoma should be performed as per the clinical condition of the patient. There is very limited data available in the existing literature regarding whether the bowel should be primarily anastomosed or a two-stage procedure should be done [1]. Vallböhmer et al. had one patient who underwent a two-stage procedure because segmental ischemia of this area developed 2 days after the repair of the diaphragmatic hernia [9]. Hiatal hernia can also be repaired via minimally invasive surgery if it presents electively. Mesh placement is usually not required and is not done especially in emergent cases. The use of mesh for the reinforcement of large hiatal hernia repairs leads to decreased short-term recurrence rates; however, there is inadequate long-term data on which to base a recommendation either for or against the use of mesh at the hiatus [14].

Another novel technique is using biologic mesh for plug repair. Narayanan et al. described the technique of repairing the diaphragmatic hiatus with a biologic mesh plug without re-approximation of the crura. This was an effective method to prevent recurrences without compromising the gastric conduit [8]. Complications directly related to diaphragm hiatus repair were few and most infections, including the empyema and pneumonia, and were more likely to occur in those who presented with acute obstruction. Preventive measures include closure of hiatus and hitching of conduit to hiatus but we do not do it in our routine practice as these measures are not standred of care as of now [7].

Significant morbidity is associated with hiatal hernia repair post-esophagectomy, with a pooled prevalence of morbidity of 35% and in-hospital mortality rate of 5%. Associated complications are pulmonary (14.1%), cardiovascular (9%), and anastomotic leak (1%). These rates are especially higher in emergency patients. The recurrence rate after surgical repair is also high (16%) in these patients, and reoperation might also be required in 12% of patients [1]. There is also significant variability in the literature regarding recurrence rates, ranging from 13% to 29%.

## Conclusions

Post-esophagectomy hiatal hernia is a rare entity with presentation ranging from asymptomatic in the immediate post-operative period or a delayed acute emergency. Significant morbidity is associated with patients presenting as an emergency. Successful management in such cases depends on prompt detection, early surgery, proper post-operative care, and rehabilitation.
